# The relationship between elastography-based muscle properties and vertical jump performance, countermovement utilization ratio, and rate of force development

**DOI:** 10.1007/s00421-023-05191-7

**Published:** 2023-04-12

**Authors:** Daniel Djurić, Jernej Pleša, Bas Van Hooren, Žiga Kozinc, Nejc Šarabon

**Affiliations:** 1 Faculty of Health Sciences, University of Primorska, Polje 42, 6310 Izola, Slovenia; 2 Department of Nutrition and Movement Sciences, NUTRIM School of Nutrition and Translational Research in Metabolism, Maastricht University Medical Centre+, Maastricht, The Netherlands; 3 Andrej Marušič Institute, University of Primorska, Muzejski trg 2, 6000 Koper, Slovenia; 4 Human Health Department, InnoRenew CoE, Livade 6, 6310 Izola, Slovenia; 5 Laboratory for Motor Control and Motor Behavior, S2P, Science to Practice, Ltd., Tehnološki Park 19, 1000 Ljubljana, Slovenia

**Keywords:** Muscle stiffness, Eccentric utilization ratio, Jumping performance, Ankle, Knee, RFD

## Abstract

This study explored the relationships between passive muscle stiffness (shear modulus) and vertical jumping performance, countermovement utilization ratio (CUR) and rate of force development (RFD) in an attempt to unravel the mechanism that may explain the association between shear modulus and performance. 32 recreationally active participants (16 males, 16 females; age: 22.4 ± 5.1 years) participated. Shear modulus was assessed for the lateral and medial gastrocnemius (GL and GM), and vastus medialis (VM) and lateralis (VL) muscles using shear wave elastography. Squat jump (SJ) and countermovement (CMJ) jump were determined, with CUR being expressed as the ratio between the two. RFD in ankle and knee extension tasks was measured using isometric dynamometers. Our results suggest that within a heterogeneous group of recreational athletes, passive muscle stiffness is not related to RFD and jump performance, but positively related to CUR. In males, shear modulus of the GL was positively related to SJ height (r = 0.55). We also found inverse moderate correlations between VL and VM shear modulus and RFD in females only (r = –0.50 to –0.51), but this relationship was possibly affected by age and body fat content. Different mechanisms may underpin the association between shear modulus and performance depending on the muscle, task and population investigated.

## Introduction

In strength and conditioning, strength and power are the most commonly tested capabilities, owing to their significant importance for sport performance (Young 2006; McMaster et al. 2014), as well as for injury risk (Faigenbaum and Myer 2010; De La Motte et al. 2017). To better understand the limiting factors of strength and power capabilities and exploit this knowledge in athletic training, researchers have investigated potential determinants such as muscle stiffness (Wilson et al. 1994; Pruyn et al. 2016). However, the majority of the previous studies have used indirect measurements of muscle stiffness, including joint-level stiffness (inferred from the relationship between joint angle and joint torque) (Günther and Blickhan 2002; Blackburn et al. 2004) or whole-leg stiffness (typically assessed as the ratio between the vertical ground reaction force and center of mass displacement within tasks involving a stretch–shortening cycle, such as drop jumps or hops) (Kyröläinen and Komi 1995; Komi 2000; Serpell et al. 2012). In such tasks, both neural and passive muscle–tendon properties may contribute to the measured stiffness, which makes it difficult to assess the relative contribution of different factors.

For a long time, direct measurements of muscle passive stiffness were only done through ex vivo mechanical testing on cadaveric muscles (Ettema 1997). However, these methods do not allow for a comprehensive insight into human muscle characteristics in vivo (Komi 1990), with factors such as muscle temperature (Nocella et al. 2013; Bernabei et al. 2020) and post-mortem stiffening potentially confounding the results. Shear-wave elastography (SWE) is an emerging method that enables muscle-specific assessment of passive stiffness in vivo through shear modulus quantification. Briefly, SWE is based on exciting a shear-wave using an acoustic push, and subsequently measuring the speed with which the shear-waves propagate across the tissue (for detailed reviews, see Blank et al. 2021; Creze et al. 2018). A faster propagation reflects a higher stiffness. SWE has high validity for estimating passive muscle stiffness, as shown in animal and human ex vivo studies (Eby et al. 2013; Kodesho et al. 2021), and also correlates with other methods for assessing muscle stiffness, such as myotonometry (Do et al. 2021). In addition, SWE can detect changes in muscle stiffness (assessed ex vivo with short-range stretch response technique) that are independent of changes in muscle tension (Bernabei et al. 2020).

The physiological mechanisms that influence passive muscle stiffness remain however largely unknown, and as a consequence, so do the implications for performance. For example, slow twitch muscle fibers (Type I) are stiffer than fast twitch muscle fibers (Type II) (Kovanen et al. 1984; Goubel and Marini 1987), which suggests that a higher passive stiffness as measured with SWE could reflect a higher proportion of type I fibers. Because type I fibers show a lower rate of force development than type II fibers, a higher shear modulus (stiffness) could therefore be associated with a lower rate of force development. In support of this mechanistic association, long-distance runners -which are expected to exhibit a relatively large proportion of type I fibers- exhibited a higher shear modulus of the vastus lateralis compared to sprinters and controls (Miyamoto et al. 2019). Shear modulus has however also been shown to exhibit a positive relationship with passive joint stiffness (Chino and Takahashi 2016) and passive muscle force (Eby et al. 2013). Given that resting passive force has been associated with an increased rate of force development (RFD) (De Ruiter et al. 2008), a higher shear modulus (stiffness) could also positively be related to RFD, and by extension to performance in sports requiring rapid force production (Laffaye and Wagner 2013; Lum and Joseph 2020). Although this positive correlation would be in contrast to the association between higher passive stiffness and RFD inferred from fiber typology, some findings also support this latter hypothesis. For example, gastrocnemius shear modulus showed a moderate positive relationship (r = 0.46–0.49) with ankle extension RFD (Ando and Suzuki 2019). A recent study extended these findings by showing that passive gastrocnemius shear modulus was positively related to plantar flexion RFD (r = 0.54–0.60) and also to 100-m sprint performance (r = 0.58) (Yamazaki et al. 2022). Moreover, gastrocnemius shear modulus also appears to be positively related to drop jump performance (r = 0.41 for jump height and reactive strength index) (Ando et al. 2021a) and another study also found the shear modulus of several lower limb muscles to be positively associated with CMJ performance (Akkoc et al. 2018).

Because both hypotheses regarding the underlying mechanism are therefore supported by indirect evidence in the literature, the physiological mechanism associated with SWE-derived passive stiffness remains equivocal. One way to obtain more insight into the physiological mechanism that explains the correlations between shear modulus and performance is by integrating findings across different muscles groups and experiments. For example, previous studies reported a strong correlation between single-joint RFD and the proportion of type II fibers (r = 0.59–0.85; Methenitis et al. 2019). Similarly, positive correlations have been reported between squat jump performance and the percentage of type II fibers (r = 0.51–0.79; Bosco and Komi 1979; Fry et al. 2003). If passive stiffness as assessed using SWE primarily reflects the proportion of type I fibers, it should therefore show a negative association with both RFD and SJ performances. On the other hand, if SWE assessments primarily reflect resting muscle tension (De Ruiter et al. 2008) or stiffness of intra-muscular connective tissues independently of fiber type (Purslow 1989), it should show a positive correlation with RFD and SJ performances. Moreover, Van Hooren and Zolotarjova, (2017) proposed that a large difference between CMJ and the SJ performance (i.e. countermovement utilization ratio (CUR)) could reflect a poor ability to rapidly develop force, increased muscle compliance (lower stiffness), and a larger amount of muscle slack that needs to be taken up in the SJ. Therefore, it could be expected that muscle stiffness would be negatively correlated with CUR. While a higher tendon stiffness has already been shown to be related to lower difference between CMJ and SJ performances (i.e., lower CUR) (Kubo et al. 1999), the relationship between CUR and passive muscle stiffness remains unexplored.

Therefore, the purpose of this study was to explore the relationships between passive shear modulus obtained through SWE and vertical jumping performance (SJ and CMJ), CUR, as well as isometric RFD in ankle and knee extension tasks. While previous studies have partially addressed these questions, they were often limited to one muscle (Ando and Suzuki 2019; Ando et al. 2021a; Yamazaki et al. 2022), with this single muscle also being measured at one joint position. Because a positive relationships exist between muscle length and shear modulus (Xu et al. 2018), it is reasonable to assume that shear modulus obtained at different muscle lengths could provide differential information. Yamazaki et al. (2022) further indicated that the relationship between RFD and shear modulus is the highest when the latter is obtained with muscles at rest (passive shear modulus), compared to measurements on active muscles (active shear modulus). The correlations with 100-m sprint performance were similar for passive and active shear modulus (Yamazaki et al. 2022). Therefore, we decided to assess only passive shear modulus, and rather compare the shear moduli and their associations with performance variables in two muscle groups (gastrocnemius and quadriceps) and also in two different joint configurations (for the quadriceps). We hypothesized that shear modulus will be negatively related to RFD (Ando and Suzuki 2019; Yamazaki et al. 2022) and possibly vertical jumping performance (Akkoc et al. 2018; Ando et al. 2021a), but inversely related to CUR (Van Hooren and Zolotarjova 2017; Kozinc et al. 2021).

## Methods

### Participants

A convenience sample of 32 healthy young participants was recruited for the study (16 males; age: 24.9 ± 3.6 years; body mass: 81.7 ± 9.7 kg; body height: 181.3 ± 7.5 cm; 16 females; age: 21.2 ± 3.5 years; body mass: 66.0 ± 7.8 kg; body height: 169.2 ± 5.7 cm). The required sample size was determined (α = 0.05; power = 0.80) based on previous studies that showed a significant moderate correlation between shear modulus, athletic performance and RFD (r =  ~ 0.4–0.5; Ando and Suzuki 2019; Ando et al. 2021a)) with 25 participants. Additional participants were recruited to prevent underpowering, as some studies with similar sample size (n = 24) failed to show a significant relationship between shear modulus and performance (Akkoc et al. 2018). The participants were all recreationally active in various sports (handball, soccer, volleyball and basketball), and reported that they were performing 3.6 ± 2.3 resistance training sessions per week in the last 6 months. The average body mass index was 23.9 ± 2.5 (24.6 ± 2.4 for males; 23.1 ± 2.3 for females). Exclusion criteria were any lower leg injuries in the past 6 months, pregnancy, any known neurological diseases, or any current musculoskeletal pain. Participants were thoroughly informed about the experimental protocol and written informed consent was required prior to commencing the study. The experiment was approved by National Medical Ethics Committee (approval no. 0120–99/2018/5) and was conducted in accordance with the latest revision of the Declaration of Helsinki.

### Study design

The participants were asked to refrain from any strenuous physical activity (i.e., resistance exercise and moderate-to-high intensity endurance activities) in 48 h before the measurement session. The experiment was conducted within a single session, lasting approximately 90 min. Upon arriving to the laboratory and signing the informed consent, the participants rested for 15 min in a prone position, after which the SWE measurements were taken. Then, the participants performed a warm-up consisting of 5 min of light-intensity running on a treadmill, 5 min of dynamic stretching (e.g., arm circles, leg swings, march and reach, trunk rotations) and a set of 5 bodyweight resistance exercises (10 squats, 5 push-ups, 5 lunges per leg, 5 submaximal countermovement jumps, 10 calf raises). The warm-up was designed to encompass the typical routines of recreational athletes, which ensures that the results of the study have the best validity in relation to typical sport conditions for these individuals. Then, the participant went on to perform (in that order) vertical jumping assessments, followed by isometric knee extension and ankle plantarflexion strength (peak torque) and explosive strength (RFD) assessments. Rest between measurement sections was 5 min. The order of the joints (knee, ankle) was randomized for each participant, while vertical jumping assessment always preceded the assessment of isometric strength and explosive strength. The experiment was carried out in an air-conditioned laboratory (23 ± 1 °C). The temperature was checked regularly using a Metrel^®^ Multinorm (model MI 6201) with an microclimatic probe (model A1091).

In addition to examining the relationships between shear modulus, jump performance, and RFD on the whole sample, we also examined a potentially confounding effect of sex, age, body mass, and height. Namely, neuromuscular performance was expected to be higher in males than females (Bassett et al. 2020), while potential sex differences in muscle shear modulus are unclear at this point (Miyamoto et al. 2018; McPherson et al. 2020). Females could also have different shear modulus values due to a higher proportion of subcutaneous adipose tissue (Yoshitake et al. 2023).

### Shear wave elastography

Participants were instructed to lay prone and relax on a physical therapy table for 15 min prior to SWE measurements. As in previous studies using SWE (Morales-Artacho et al. 2017), this was done to minimize the possible confounding effects of prior activities (e.g., walking) on muscle shear modulus. The ankles were positioned over the edge of the table to ensure that the ankle was in neutral position. Then, we first obtained the shear modulus for medial (GM) and lateral (GL) head of the gastrocnemius, followed by the measurements for vastus medialis (VM) and vastus lateralis (VL). For the GM and GL assessments, the participant remained in the relaxed prone position and the probe was positioned on an imaginary line spanning from the heel to the medial and lateral condyles, respectively, on the most prominent aspect of the muscles. For VM and VL, the participants switched to supine position. For the VM, the probe was placed at distance 80% on the line between the anterior spina iliaca superior and the joint space in front of the anterior border of the medial collateral ligament. For VL, the probe was placed at 2/3 on the line from the anterior spina iliaca superior to the lateral side of the patella. For all muscles, the positions were carefully determined and the position marked with a non-permanent skin marker. For the VM and VL, we first performed the measurements at an extended knee position (knee angle 0°), followed by additional assessment at a flexed knee position (knee angle 90°). For the latter, the participant’s limb was moved to the appropriate position by the examiner to avoid muscle activation, and the knee angle was verified with a manual goniometer. Before SWE measurements, the B-mode ultrasound was used to ensure the parallel alignment with the direction of the muscle fascicles. A probe orientated in parallel to the muscle fascicles has been demonstrated to provide the most reliable and valid muscle stiffness assessment (Gennisson et al. 2010; Eby et al. 2013).

**Fig. 1:**
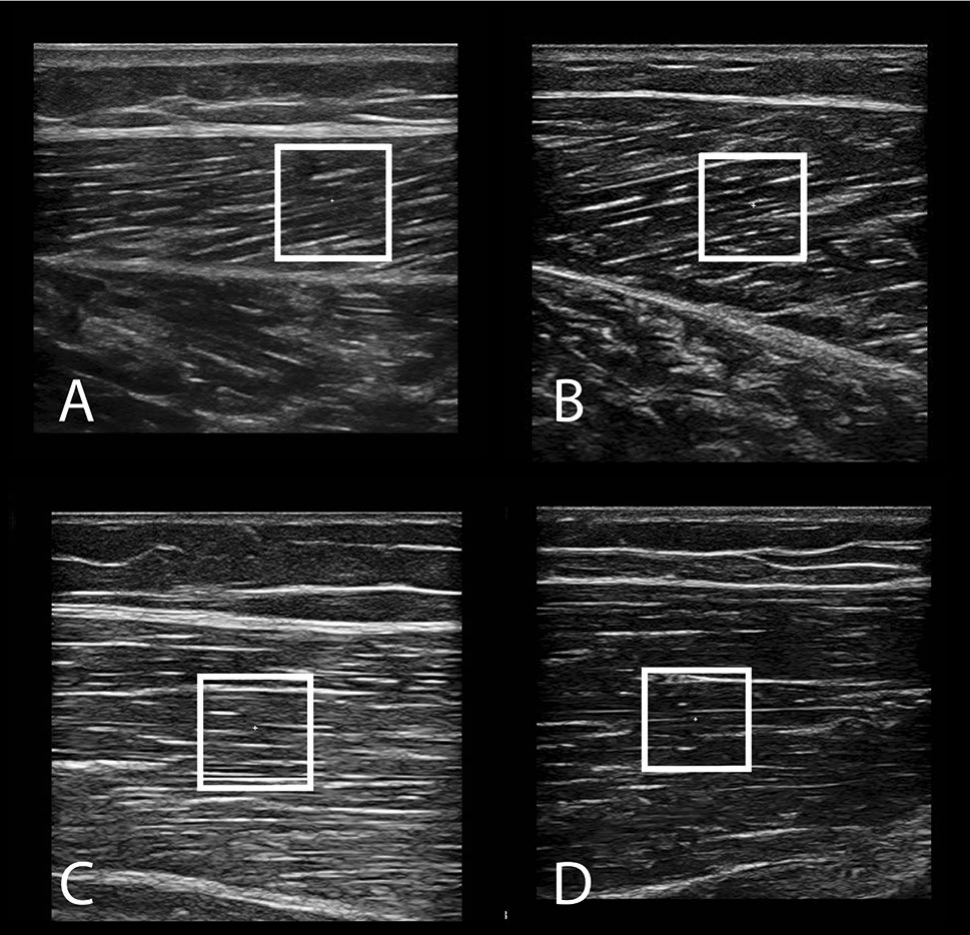
Ultrasound image samples for lateral (**A**) and medial (**B**) head of the gastrocnemius, vastus lateralis (**C**) and vastus medialis (**D**) muscles. The white square represents the region of interests for shear modulus quantification

We used the Resona 7 ultrasound system (Mindray, Shenzhen, China), set to musculoskeletal mode, which assumes a tissue density of 1000 kg/m^3^. A 4-cm linear array probe (3–11 MHz; Model L11-3U, Mindray, Shenzhen, China) with a generous amount of water-soluble hypoallergenic ultrasound gel (AquaUltra Basic, Ultragel, Budapest, Hungary) was used. The region of interest was fixed at a square shape of 1 cm^2^. The depth of the region of interest was left to the choice of the examiner, with special care taken to only include muscle tissue. One measurement of the shear modulus assessment took about 8 s. The device takes 8 consecutive scans and displays the mean value. If the variability of the values within the 8 scans was above 15%, the measurement was repeated. In total, three measurements, with 1 min breaks in between, were performed and the mean value was taken for further analyses. Snapshots of ultrasound images are shown in Fig. 1.

### Vertical jumping assessment

For each jump task (SJ and CMJ), the participants performed 2–3 attempts with submaximal effort, after which 3 repetitions were recorded for each jump in alternating order. Rest periods were set at 1 min between repetitions. For both jumps, the hands were placed on the hips throughout the entire trial. SJ was performed with the participant descending to the starting position which was achieved when the knee angle was at 90°. After stabilizing for 2–3 s, the jump was performed without countermovement. The examiner zoomed in and visually inspected the force–time trace on the computer and the trial was repeated in case any perceivable countermovement was detected (Pérez-Castilla et al. 2019; Suchomel et al. 2020; Janicijevic et al. 2022). For the CMJ, the participants were instructed to jump as high as and as fast as possible, using a countermovement (to the point where the knee angle was at 90°) and then immediately pushing off and extending forcefully through the hips, knees and ankles. Before each jump, participants were required to squat in a controlled manner until the desired position was reached to become familiarized with the 90° knee angle position, which was previously determined with a manual goniometer. In addition, an elastic band was positioned for each individual on the appropriate height to be in contact with participants’ buttocks when the desired angle was reached in SJ. For the CMJ, the elastic band was moved away from participants to avoid contact during the jump, and one examiner stood nearby the participant to visually control that the appropriate CMJ depth was reached (Janicijevic et al. 2022).

Ground reaction force data was captured with single force plate (Kistler, model 9260AA6, Winterthur, Switzerland). The signals were further automatically processed by the manufacturer’s software (MARS, Kistler, Winterthur, Switzerland) with a moving average filter (window: 5 ms). The vertical acceleration of the participant’s CoM was calculated via Newton’s second law of motion (F = m·a); subsequently, the acceleration was integrated to obtain vertical velocity using the trapezoid rule. Jump height was calculated from the takeoff velocity (TOV) as follows:

$$\mathrm{Jump}\, \mathrm{height}=\frac{{\mathrm{TOV}}^{2 }}{2g}$$

Were *g* is the acceleration of gravity (9.81 m∙s^−2^).

The best repetition (i.e., highest jump height) was taken for further analyses. CUR was calculated as follows: CUR = [CMJ-SJ]/SJ * 100%.

### Isometric strength and explosive strength assessment

**Fig. 2:**
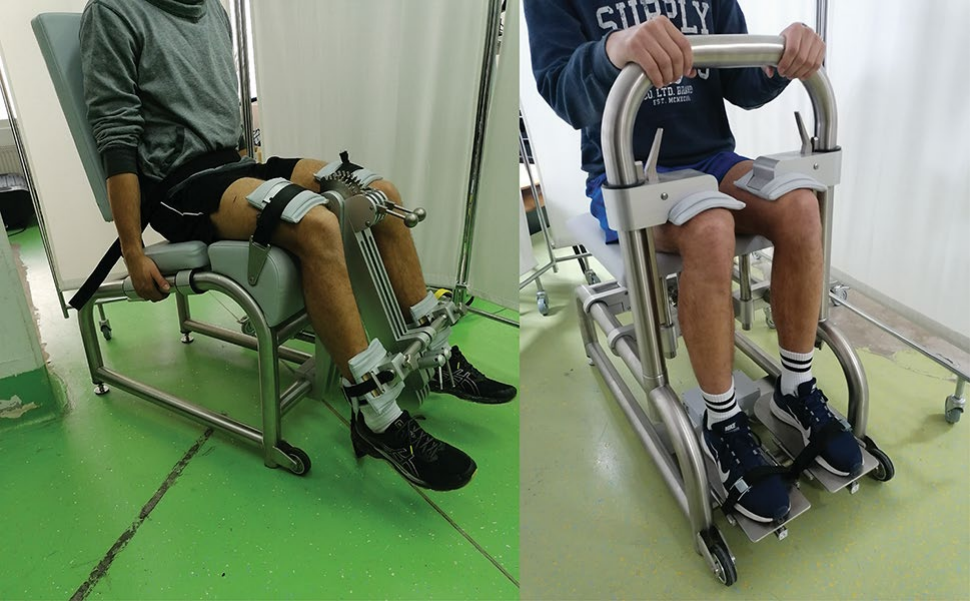
Participant positioning for knee (left) and ankle (right) isometric strength assessments

All isometric strength and RFD assessments were conducted using isometric dynamometers (S2P, Science to Practice, Ljubljana, Slovenia) with embedded force sensors (model 1-Z6FC3/200 kg) (Fig. 2). For the assessment of the ankle extension, the participant’s shins were tightly secured within the dynamometer frame, and the feet were placed on a rigid metal plate mounted on the force sensor. The axis of the dynamometer was carefully aligned with the medial malleolus and the ankle was in the neutral position (90°). The foot was tightly fixated against the plate with a strap. The knee and hip angles were set at 90° as well, which was achieved by adjusting the height and the depth of the dynamometer seat. For the assessment of the knee extension, the participants were seated in the dynamometer, tightly fixated across the pelvis and thighs, with the axis of the dynamometer aligned to the lateral femoral condyle. The knee angle was set to 60° (0° = full knee extension) and the hip was flexed at 90°. The instruction to the participants was to produce the force as “fast and as hard as possible”, and to maintain maximal exertion for ~ 3–4 s. The participants were instructed and constantly reminded to focus on emphasizing the explosive start (the fast part of the contraction). Three repetitions with 2 min breaks in between were performed. Loud verbal encouragement was provided at all times and the participants received real-time feedback on force trace on a computer screen.

The force signals were sampled at 1000 Hz and were further automatically processed in the manufacturer’s software (Analysis and Reporting Software, S2P, Ljubljana, Slovenia). A moving average filter (window: 5 ms) was applied, after which the RFD was calculated as Δ force /Δ time for 0–50, 0–100, 0–150 and 0–200 ms time windows. To ensure an accurate determination of force rise onset, we used custom-made software for semi-manual analysis (Maffiuletti et al. 2016). The instance of onset of torque rise was automatically detected by the software as the instant at which the baseline signal exceeded 3% of the peak torque. This was shown by a vertical marker placed on the torque-time trace. The examiner then zoomed in and moved the marker to more accurately determine the instant of force rise. Peak torque was determined as the mean value of the 1 s interval. For all of the outcome variables, the best repetition (i.e., highest value) was considered for the main statistical analyses (differences and correlations).

### Statistical analysis

Statistical analyses were done with SPSS (version 25.0, SPSS Inc., Chicago, IL, USA). Descriptive statistics are reported as mean ± standard deviation. The normality of the data distribution was verified with a Shapiro–Wilk tests and visual inspection of Q-Q plots and histograms. The rep-by-rep reliability of outcome measures was assessed with the ICC (two-way random, absolute agreement model) and typical error, which was calculated as the standard deviation of the absolute differences between repetitions, divided by the square root of 2 (Hopkins 2000). Typical errors were expressed as % of the mean value (i.e., coefficient of variation; CV). The reliability according to ICC was interpreted as poor (< 0.5), moderate (0.5–0.75), good (0.75–0.9) and excellent (> 0.9) (Koo and Li 2016). The reliability according to CV was considered as excellent (< 5%), good (5–10%), acceptable (10–15%) and unacceptable (> 15%) (Shechtman 2013). Differences between males and females were examined with an independent-sample t-test. The associations between the outcome variables were assessed with Pearson’s product-moment correlations and interpreted as negligible (< 0.1), weak (0.1–0.39), moderate (0.4–0.69), strong (0.7–0.89) and very strong (> 0.9) (Schober and Schwarte 2018).We used a Benjamini–Hochberg method to control for the false discovery rate due to multiple correlations (Benjamini and Hochberg 1995). The threshold for statistical significance was set at α < 0.05.

## Results

### Reliability

The relative reliability of shear modulus outcomes was good to excellent (ICC = 0.88–0.94). CVs were slightly above 10% for GL and VM when then knee was extended (10.5–12.1%), but below 10% for the remaining outcomes (5.8–9.8%). For both ankle and knee extensors RFD, the relative reliability was excellent for RFD_150_ and RFD_200_ (ICC = 0.90–0.94), good for RFD_100_ (ICC = 0.82–0.87) and moderate to good for RFD_50_ (ICC = 0.62–0.78). CVs decreased with the time interval of the RFD, but were below 10% only for the RFD_150_ and RFD_200_ for the ankle (7.3–9.4%) and RFD_200_ for the knee (8.8%). SJ height, CMJ height and CUR exhibited good to excellent reliability (ICC = 0.85–0.98) with acceptable CVs (2.9–8.8%).

### Descriptive statistics and sex differences

The shear modulus values were not statistically significantly correlated between extended and flexed knee positions for VM (r = 0.31 p > 0.562). For VL, there was a moderate and statistically significant correlation between shear modulus in extended and flexed knee positions (r = 0.51; p < 0.002).

Table 1 contains the descriptive statistics and t-test outcomes used to assess sex differences. There were no sex differences in shear modulus for any muscle (p ≥ 0.122). Males had higher peak torque for both ankle extension (p = 0.031; d = 0.39) and knee extension (p < 0.001; d = 0.70). While males also had higher RFD for the knee extension (p = 0.001–0.017; d = 0.44–0.64) there were no sex differences for ankle extension RFD (p = 0.789–0.945). Males also presented larger jump heights in SJ and CMJ (p < 0.001; d = 1.38 for SJ and 1.57 for CMJ). Males also had larger CUR (p = 0.046; d = 0.36). These differences, coupled with higher body mass and body height in males, justified sex-specific correlation analysis.

**Table 1:** Descriptive statistics and sex differences

Outcome measure	Male		Female		Difference		
	Mean	SD	Mean	SD	t	p	d
SM (GL) (kPa)	10.96	4.26	11.38	3.36	− 0.30	0.760	− 0.05
SM (GM) (kPa)	9.93	3.55	10.51	1.60	− 0.60	0.551	− 0.10
SM (VL-knee extended) (kPa)	7.69	2.76	6.93	2.46	0.84	0.405	0.15
SM (VM-knee extended) (kPa)	7.62	2.44	7.21	2.12	0.51	0.610	0.09
SM (VM-knee flexed) (kPa)	12.28	1.99	11.23	1.80	1.58	0.122	0.28
SM (VL-knee flexed (kPa)	12.69	3.36	11.48	1.68	1.29	0.204	0.23
Ankle peak torque (Nm/kg)	4.60	0.60	4.07	0.76	2.26	**0.031**	0.39
Ankle RFD50 (Nm/kg/s)	15.28	6.28	15.65	4.17	− 0.19	0.847	− 0.03
Ankle RFD100 (Nm/kg/s)	19.09	6.43	19.46	4.60	− 0.19	0.848	− 0.03
Ankle RFD150 (Nm/kg/s)	18.21	5.15	18.65	4.09	− 0.27	0.789	− 0.05
Ankle RFD200 (Nm/kg/s)	16.41	4.07	16.32	3.57	0.06	0.945	0.01
Knee peak torque (Nm/kg)	8.81	1.61	6.58	1.58	4.00	**0.000**	0.70
Knee RFD50 (Nm/kg/s)	49.16	16.05	34.84	16.63	2.51	**0.017**	0.44
Knee RFD100 (Nm/kg/s)	45.50	10.05	34.16	10.69	3.14	**0.004**	0.55
Knee RFD150 (Nm/kg/s)	38.96	8.35	28.84	8.01	3.54	**0.001**	0.62
Knee RFD200 (Nm/kg/s)	32.64	6.78	24.25	6.31	3.67	**0.001**	0.64
SJ height (m)	0.38	0.05	0.26	0.03	7.91	**0.000**	1.38
CMJ height (m)	0.42	0.05	0.28	0.03	9.04	**0.000**	1.57
CUR jump height	1.11	0.06	1.06	0.06	2.08	**0.046**	0.36

Statistically significant differences are highlighted in bold

*SD* standard deviation, *d* effect size (Cohen’s d), *SM* shear modulus, *GL* gastrocnemius laterialis, *GM* gastrocnemius medialis, *VL* vastus lateralis, *VM* vastus medialis, *RFD* rate of force development, *SJ* squat jump, *CMJ* countermovement jump, *CUR* countermovement utilization ratio

### Correlations between shear modulus and vertical jumping performance

There were no correlations between shear modulus of any muscle and SJ (|r|= 0.02–0.21), nor CMJ height (|r|= 0.02–0.25). VL shear modulus in extended knee position (r = 0.46; p = 0.008) and VM shear modulus in flexed knee position (r = 0.39; p = 0.041) positively correlated with CUR.

In males, the shear modulus of the GL was positively related to SJ height (r = 0.55; p = 0.023). In males, the shear modulus of VM and VL (both in relaxed and stretched states) was positively related to CUR (r = 0.54–0.64; p = 0.010–0.034). No statistically significant associations were found in females (Table 2).

**Table 2:** Correlations between muscle shear modulus with jumping performance and countermovement utilization ratio

	Shear modulus					
	GL	GM	VM (KE)	VL (KE)	VM (KF)	VL (KF)
Whole sample						
SJ height	0.21	− 0.02	0.09	− 0.07	0.16	− 0.02
CMJ height	0.10	0.02	0.18	0.03	0.25	0.08
CUR	− 0.35	0.15	0.34	0.46**	0.39*	0.34
Males						
SJ height	0.55*	0.11	− 0.05	− 0.39	0.04	− 0.40
CMJ height	0.39	0.19	0.19	− 0.18	0.27	− 0.20
CUR	− 0.46	0.19	0.62**	0.59*	0.59*	0.54*
Females						
SJ height	0.22	0.22	− 0.06	− 0.02	− 0.42	− 0.24
CMJ height	0.03	0.34	− 0.10	0.09	− 0.43	− 0.33
CUR	− 0.45	0.25	− 0.05	0.29	− 0.05	− 0.19

*GL* gastrocnemius laterialis, *GM* gastrocnemius medialis, *VL* vastus lateralis, *VM* vastus medialis, *SJ* squat jump, *CMJ* countermovement jump, *CUR* countermovement utilization ratio, *KE* with knee extended, *KF* with knee flexed

*p < 0.05

**p < 0.01

### Correlations between shear modulus and rate of force development

There were no significant correlations between shear modulus of any muscle and RFD when the whole sample was analyzed (|r|< 0.27; p ≥ 0.089). The details are available in Table 3.

**Table 3:** Correlations between muscle shear modulus with rate of force development outcomes

	Shear modulus					
	GL	GM	VM (KE)	VL (KE)	VM (KF)	VL (KF)
Whole sample						
Ankle peak torque	0.04	0.11	0.00	− 0.08	0.01	− 0.03
Ankle RFD50	0.20	− 0.12	0.08	− 0.10	− 0.10	− 0.10
Ankle RFD100	0.16	− 0.10	0.02	− 0.20	− 0.09	− 0.18
Ankle RFD150	0.15	− 0.07	− 0.02	− 0.22	− 0.04	− 0.21
Ankle RFD200	0.16	− 0.04	− 0.04	− 0.27	− 0.03	− 0.21
Knee peak torque	0.07	− 0.03	0.07	− 0.02	0.06	0.03
Knee RFD50	− 0.15	− 0.18	0.05	− 0.08	0.04	− 0.08
Knee RFD100	− 0.09	− 0.11	0.11	− 0.08	0.09	0.01
Knee RFD150	− 0.07	− 0.10	0.08	− 0.10	0.07	0.02
Knee RFD200	− 0.05	− 0.07	0.07	− 0.06	0.02	0.01
Males						
Ankle peak torque	− 0.05	0.23	− 0.19	0.04	0.19	− 0.05
Ankle RFD50	0.20	− 0.18	0.17	0.12	− 0.08	− 0.12
Ankle RFD100	0.10	− 0.18	0.11	− 0.02	− 0.06	− 0.21
Ankle RFD150	0.03	− 0.14	0.09	− 0.03	0.04	− 0.24
Ankle RFD200	0.03	− 0.11	0.05	− 0.07	0.07	− 0.26
Knee peak torque	0.13	0.10	− 0.03	− 0.02	0.08	− 0.05
Knee RFD50	− 0.39	− 0.26	0.08	− 0.11	0.12	− 0.07
Knee RFD100	− 0.26	− 0.13	0.16	− 0.04	0.20	0.07
Knee RFD150	− 0.20	− 0.11	0.08	− 0.13	0.12	0.03
Knee RFD200	− 0.12	− 0.07	0.03	− 0.13	0.10	0.01
Females						
Ankle peak torque	0.20	0.10	0.07	− 0.29	− 0.38	− 0.28
Ankle RFD50	0.18	0.06	− 0.06	− 0.51*	− 0.12	− 0.01
Ankle RFD100	0.28	0.11	− 0.12	− 0.49	− 0.12	− 0.10
Ankle RFD150	0.35	0.12	− 0.17	− 0.50*	− 0.12	− 0.12
Ankle RFD200	0.36	0.14	− 0.15	− 0.47	− 0.18	− 0.14
Knee peak torque	0.10	− 0.07	0.00	− 0.17	− 0.39	− 0.36
Knee RFD50	0.18	0.05	− 0.12	− 0.14	− 0.32	− 0.47
Knee RFD100	0.16	0.05	− 0.10	− 0.27	− 0.33	− 0.51*
Knee RFD150	0.18	0.10	− 0.11	− 0.25	− 0.37	− 0.48
Knee RFD200	0.13	0.13	− 0.06	− 0.13	− 0.50*	− 0.50*

*GL* gastrocnemius laterialis, *GM* gastrocnemius medialis, *VL* vastus lateralis, *VM* vastus medialis, *RFD* rate of force development, *KE* with knee extended, *KF* with knee flexed

*p < 0.05

In females, ankle RFD50, RFD100 and RFD150 were inversely related to shear modulus of VL in relaxed state (r = –0.50 to –0.51; p = 0.047–0.049). Knee RFD200 was inversely related to shear modulus of VL and VM in the stretched state (r = –0.50; p = 0.048). Knee RFD100 was also inversely related to shear modulus of VL in the stretched state (r = –0.51; p = 0.048). In males, no statistically significant associations among shear modulus and RFD variables were found (Table 3).

### Association between shear modulus and body mass index, body mass, body height and age

We assessed the correlations between age and shear modulus and body mass index, body mass, body height and age, as well as conduct partial correlations controlling for these variables. The results were mainly unchanged. Shear modulus values were not associated with body mass index, body mass, body height or age of the participants when considering the whole sample (r <|0.25|; p > 0.152), males only (r <|0.33|; p > 0.212) and females only (r <|0.35|; p > 0.074). The only exception was an inverse correlation between age and shear modulus of the VL in the extended knee position (r = –0.53; p = 0.036) in females only. A partial correlation controlling for age in females reduced the correlation coefficients between VL shear modulus and knee RFD in females (r = –0.48 to 0.51 without controlling for age; r = –0.30 to –0.41 with controlling for age; p = 0.112–203), which suggest that the relationship between shear modulus and knee RFD is, at least partially, explained by age. Indeed, there was also a positive, but a non-significant correlation between age and knee RFD in females (r = 0.41–0.43; p = 0.053–0.151).

## Discussion

This study aimed to investigate the relationships between passive shear modulus of various lower limb muscles, and vertical jumping performance (SJ and CMJ), CUR and single-joint RFD in an attempt to gain further insight into the physiological mechanism that might explain the relationship between passive shear modulus and performance observed in previous studies. We hypothesized that shear modulus would be positively related to RFD and vertical jumping performance, but inversely related to CUR. Our first and second hypothesis were not confirmed, as we found no correlations between shear modulus and RFD, nor between shear modulus and jumping performance. Our third hypothesis was also not supported; contrary to our expectations, we found positive correlations between CUR and shear modulus of the upper leg (VL and VM) muscles.

Two other studies recently observed a positive relationship between GM shear modulus and isometric plantarflexion RFD (Ando and Suzuki 2019; Yamazaki et al. 2022), while Ema (2022) reported a positive association between VL shear modulus and power output during a fast, but not slow leg press task in 30 healthy women. Contrary to these findings, we observed no associations between GM and GL shear modulus and plantar flexion RFD. We also found no correlation between VL and VM shear modulus and knee extensor RFD. The lack of correlation in our study could be explained by the sample of participants. Specifically, our sample was relatively heterogenous and variations in fiber type composition could therefore have influenced the correlations between shear modulus and RFD. In this context, slow twitch (type I) muscle fibers are stiffer than fast twitch muscle fibers (Kovanen et al. 1984; Goubel and Marini 1987), and a higher shear modulus could therefore also reflect a higher proportion of type I fibers, which have a slower RFD than type IIa or type IIx fibers. Yet, it could be that within a homogenous group of athletes (e.g., sprinters with a comparable proportion of type II and I fibers) a higher shear modulus is beneficial because it reflects higher stiffness of the predominantly fast twitch fibers, which would in turn enhance the RFD (as reported by Yamazaki et al. (2022)). Within heterogenous groups, the variability in shear modulus may therefore be partially explained by the variability in muscle fiber composition, while a different mechanism may explain the variability of shear modulus within homogeneous groups (e.g., elite athletes). It must be emphasized that the influence of fibre type on the relationship between passive shear modulus and performance is purely speculative at this point because we did not directly examine muscle fibre composition by muscle biopsies. Finally, intra-muscular adipose tissue content has also recently been shown to be associated with lower shear modulus (Yoshitake et al. 2023), which could further confound the correlations in the present study. Our study also points out to the importance of controlling for confounding variables, such as age and body composition. While an inverse relationship between muscle stiffens and RFD was indicated in females, it was found that this could be partially explained by age, as younger participants had lower RFD but higher muscle stiffness. The latter, in turn, could be related to body fat content (Yoshitake et al. 2023), as age was slightly (but not significantly) correlated with body mass index in females (r = 0.38; p = 0.115). Future studies should take into account the potential confounders, while the results of existing studies should be interpreted with caution.

Our results showed no association between the shear modulus and jumping performance, with only one statistically significant moderate association found in males. We hypothesized that stiffer muscles and tendons could enhance jump height due to faster force transmission and RFD (Maffiuletti et al. 2016). In support of this, individuals with stiffer patellar and Achilles tendons have slightly (~ 2–3 cm) higher SJ (and CMJ) heights than individuals with more compliant tendons (Kubo et al. 1999, 2007). However, RFD was not related to shear modulus, and we did not assess tendon stiffness, which limits the robustness of our conclusions. Contrary to our findings, a previous study also reported a positive association between gastrocnemius shear modulus and drop jump performance (Ando et al. 2021a). Interestingly, gastrocnemius shear modulus however decreased after a drop-jump training intervention (Ando et al. 2021b), without concomitant changes in RFD. Considering the participants in that study were not trained athletes, the authors suggested that drop jump training could enforce lengthening of muscles fascicles, which could result in decreased shear modulus at a given muscle length by altering the length-tension relationship and thus leading to less passive tension at a given muscle length (Brughelli and Cronin 2007). The associations between shear modulus and jumping performance may be significantly affected by the length-tension relationship and the associated muscle slack length, which were not measured in the present study (Van Hooren and Zolotarjova 2017).

Our findings however showed a positive association between the shear modulus in VL and VM muscles and CUR. Van Hooren and Zolotarjova (2017) argued that the difference between CMJ and SJ performances may reflect factors such as the ability to rapidly develop force in the SJ, and the magnitude of slack that needs to be taken up in the SJ. A larger magnitude of slack may be associated with a lower passive tension (Herbert et al. 2015), and therefore we hypothesized that a lower shear modulus would be associated with a higher CUR. However, we observed an opposite relationship, which may be because the shear modulus does not directly correspond with the slack length. Rather, the positive correlation between VL and VM shear modulus could also reflect a higher proportion of type I fibers that limited the ability to rapidly develop force in the SJ, and in turn lead to a larger CUR.

Overall, our findings indicate that there is no consistent relationship between shear modulus and performance in heterogenous group of recreational athletes, suggesting the passive muscle shear modulus does not reflect a single physiological mechanism across different tasks and muscles in a group of heterogeneous individuals.

Some limitations of the present study need to be acknowledged. First, intra-muscular adipose tissue content, which has been recently shown to correlate with shear modulus (Yoshitake et al. 2023), was not measured. Moreover, while most studies only assessed the shear modulus of typically one or two muscles, the four muscles analyzed in our study are still a small number compared to the many muscles involved in jumping. Yet, the VL and VM are arguably among the most important muscles contributing to knee extension, while the GM and GL also have an important role in jump performance by transferring energy from the knee to the ankle joint. However, we did not consider the soleus muscle, which is the most important contributor to passive and active plantar flexion when the knee is in a flexed position (Arndt et al. 1998; Vigotsky et al. 2020). This is particularly problematic with respect to RFD assessment performed with the knee flexed (90°). Therefore, the relationships between ankle RFD and stiffness of GM, GL as well as the soleus need to be further explored. Moreover, shear modulus was assessed in a relaxed state (i.e., passive shear modulus) and may therefore not correlate well with active muscle stiffness due to changes in the muscle geometry during contraction. However, this decision was motivated by findings that showed the correlations with RFD are higher for passive than active shear modulus, and the correlations with 100-m sprint performance were similar for passive and active shear modulus (Yamazaki et al. 2022). Further research is therefore required to investigate the association between both passive and active shear modulus and the inclusion of both passive and active assessments in relation to performance. We instructed the participants to completely relax during SWE assessments; however, EMG should be used in future studies to control for muscle relaxation. The shear modulus was assessed only over a small region of each muscle. However, region-specific stiffness within a muscle have been shown with SWE (Ewertsen et al. 2016), and these could impact the observed correlations. Future studies should therefore also consider investigating region-specific differences and associations with performance. Finally, the comparison to previous study is limited because the used ultrasound systems used were from different manufactures. Previous studies have used systems such as Aixplorer (Yamazaki et al. 2022) and ACUSON (Ando and Suzuki 2019), which were both recently shown to produce consistently lower shear modulus values as well as smaller variation in shear modulus compared to the system used in the present study (Wang et al. 2022). In particular the larger variation in our system could have impacted the observed correlations, and further research is clearly needed to understand how ultrasound device choice affects the relationship between shear modulus, jumping performance and RFD.

## Conclusions

This study suggests that within a heterogeneous group of recreational athletes, muscle shear modulus is not related to vertical jump performance (SJ and CUR) nor explosive strength (RFD). The conflicting findings in regard to previous studies suggests that the association between passive muscle shear modulus and performance outcomes depends on the sample homogeneity, and possibly on muscle and task investigated. Further studies are needed to reveal the relationship between shear modulus and athletic performance in more homogenous groups of athletes, and to elucidate the underlying mechanisms for the observed relationships with direct measures of potential physiological mechanisms.

## Data Availability

The data that support the findings of this study are available from the corresponding author, NŠ, upon reasonable request.
